# Finding the traces of behavioral and cognitive processes in big data and naturally occurring datasets

**DOI:** 10.3758/s13428-017-0874-x

**Published:** 2017-04-19

**Authors:** Alexandra Paxton, Thomas L. Griffiths

**Affiliations:** 1 University of California, Berkeley, Institute of Cognitive and Brain Sciences, 3210 Tolman Hall #1650, Berkeley, CA 94720-1650 USA; 2 Berkeley Institute for Data Science, University of California, Berkeley, Berkeley, CA USA; 3 Department of Psychology, University of California, Berkeley, Berkeley, CA USA

**Keywords:** Big data, Naturally occurring datasets, Open science, Online experiments, Data on the Mind

## Abstract

Today, people generate and store more data than ever before as they interact with both real and virtual environments. These digital traces of behavior and cognition offer cognitive scientists and psychologists an unprecedented opportunity to test theories outside the laboratory. Despite general excitement about big data and naturally occurring datasets among researchers, three “gaps” stand in the way of their wider adoption in theory-driven research: the *imagination* gap, the *skills* gap, and the *culture* gap. We outline an approach to bridging these three gaps while respecting our responsibilities to the public as participants in and consumers of the resulting research. To that end, we introduce Data on the Mind (http://www.dataonthemind.org), a community-focused initiative aimed at meeting the unprecedented challenges and opportunities of theory-driven research with big data and naturally occurring datasets. We argue that big data and naturally occurring datasets are most powerfully used to supplement—not supplant—traditional experimental paradigms in order to understand human behavior and cognition, and we highlight emerging ethical issues related to the collection, sharing, and use of these powerful datasets.

Humans have always left traces of our behavioral and cognitive processes. These traces have evolved with us: Where our ancestors left stone tools and cave drawings, we now leave digital traces—social media posts, uploaded images, geotags, search histories, and video game activity logs. As in the past, these traces are left both voluntarily and involuntarily. With the explosion of social media, we share more about ourselves—in more public forums and in more varied media—than ever before. At the same time, companies and governments are tracking our activity in physical and online spaces; although these data are still often proprietary, they are increasingly being shared in the interest of transparency and open science (e.g., Arribas-Bel, 2014; Domingo, Bellalta, Palacin, Oliver, & Almirall, 2013).

Our voluntary and involuntary digital traces are being mined for a variety of purposes. For example, companies use these traces to target marketing (e.g., Brown, Chui, & Manyika, 2011), computer scientists use them to improve machine learning (e.g., Hoi, Wang, Zhao, & Jin, 2012), and governments use them to allocate resources (e.g., Domingo et al., 2013). Cognitive scientists and psychologists have not yet entirely embraced these data, but doing so offers the potential to significantly further theory-driven understanding of human behavior and cognition.

Following in the footsteps of earlier calls to action (e.g., Goldstone & Lupyan, 2016; Griffiths, 2015; Jones, 2016b), we here present an overview of the unprecedented opportunities and challenges presented by these digital traces. Rather than a definitive map, we present this article as a signpost that may inspire others to forge ahead. As part of this effort, we here introduce Data on the Mind (http://www.dataonthemind.org), a new community resource for cognitive scientists and psychologists interested in using these digital traces to understand behavior and cognition. We hope to provide a practical guide to some of the biggest issues and opportunities at the intersection of theory-building research and new sources of data.

## The data revolution: Big data and naturally occurring datasets

The term *big data* first emerged in the late 1990s (e.g., Cox & Ellsworth, 1997), but it took about a decade for the concept to enter the public and scientific imagination (e.g., Campbell, 2008; Cukier, 2010). Over its lifespan, the term has encompassed a variety of meanings. Its earliest meaning is perhaps its most evocative one—“big data” as simply data too large to be worked with on a single commercial computer (Cox & Ellsworth, 1997). This remains one of the most common definitions, especially in lay use.

We prefer IBM’s “Four V’s” (IBM, 2013) of big data. The Four V’s provide dimensions along which big data can vary that can be distilled into four questions about any given dataset:

- Volume: How *much* data are included?
- Variety: How many *different kinds or sources* of data are included?
- Velocity: How *quickly* are the data able to be gathered, processed, and analyzed?
- Veracity: How *faithfully* do the data capture what they are believed to capture?

The Four V’s encourage us to consider *rich* data—not just *big* data. Importantly, the concerns of the Four V’s have natural analogues to the concerns of traditional research in cognitive science and psychology. *Volume* aligns with sample sizes. *Variety* parallels convergent validity. *Velocity* might be analogous to the research pipeline or even replication, and *veracity* clearly mirrors external validity. Viewed in this light, the concept of big data becomes more recognizable and conceptually tractable to cognitive scientists and psychologists than many might initially believe.

A related focus on the utility of *naturally occurring data* has begun to take hold in cognitive science and psychology (e.g., Goldstone & Lupyan, 2016; Jones, 2016a), although this focus has a longer tradition in other fields (e.g., economics; see chap. 1 of Davis & Holt, 1993) and in specific areas of linguistics (e.g., discourse analysis; for a review, see Speer, 2002). Naturally occurring data sets (NODS) might be called “wild” data, typically gathered as observations of people, behaviors, or events by nonscientists for nonscientific, nonexperimental purposes (but not always; cf. Goldstone & Lupyan, 2016). Although NODS are often larger than traditional experimental datasets (except, perhaps, those gathered in cognitive neuroscience), they can reasonably be on the order of mega- or gigabytes of data, rather than tera- or petabytes.

Within this space, we are chiefly interested in datasets that were not collected for experimental purposes but could—with a little creativity and the right tools—provide insight into cognition and behavior. The *volume* of these datasets is less important than their *veracity* and *variety*, although we are interested in datasets that are considered at least medium-sized. For brevity, we will call these data simply *BONDS*—**b**ig data **o**r **n**aturally occurring **d**ata **s**ets.

BONDS shine when they are used as a complement to traditional laboratory paradigms (e.g., Griffiths, 2015; Jones, 2016b). Together, lab research and BONDS form a virtuous cycle of scientific discovery. Refined experimental paradigms generate theories about human behavior and cognition, which can be tested in the real world using BONDS. BONDS can then be used to refine theories or suggest new alternatives, which can be tested in controlled lab experiments. Put simply, BONDS should *supplement*—not *supplant*—the tight experimental control of rigorous lab research.

### Examples of research with BONDS

Before moving on, we first give three concrete examples of successful, theory-driven research using BONDS. These studies have drawn from laboratory findings about human behavior and cognition to test their explanatory power in real-world data.

#### Skill acquisition in online games (Stafford & Dewar, 2014)

Stafford and Dewar used BONDS to explore skill acquisition in a naturalistic environment. The researchers investigated the relation of practice and performance in an online game (Axon; http://axon.wellcomeapps.com). Focusing on over 45,000 individuals who had played the game at least nine times over a two-month period, Stafford and Dewar were able to quantify the effects of practice and exploration-versus-exploitation strategies on player scores over time. The results confirmed previous experimental findings: Practice improved performance; the best players started with the highest scores and improved more quickly; and early exploration of game strategies correlated with better later performance. In keeping with open science practices, the researchers also published the code and data for their analyses.

Previous work on practice and performance had tended to focus either on laboratory environments (which create artificial external pressures and motivations) or on individuals who were already experts (which cannot account for differences between those who become experts and those who drop out along the way). Stafford and Dewar’s (2014) use of online gaming data not only provided an unprecedentedly large sample but also captured natural, internally motivated behavior in ways that would be difficult—if not impossible—to study in the lab.

#### Cognitive bias in purchasing behavior (Lacetera, Pope, & Sydnor, 2012)

Lacetera and colleagues used economic BONDS to understand the real-world impact of heuristics. Specifically focusing on the *left-digit bias*, the researchers analyzed over 22 million used-car sales to investigate how the 10,000s digit (i.e., the leftmost digit) on odometers (i.e., the number of miles that a car had been driven) affected the purchase price. These data quantified the cost of purchaser inattention, finding that buyers were much more sensitive to differences in mileage across left-digit boundaries (e.g., 79,900–79,999 vs. 80,000–80,099) than to identical differences in mileage within the same left-digit boundary (e.g., 79,900–79,999 vs. 78,900–78,999). In doing so, the authors were able to confirm theory predictions about purchasers’ general inattention to details and the corresponding use of left-hand digits as cues. Although the authors make their entire dataset available only upon request, they do freely provide data analysis files with the article.

Although it was framed in terms of a question in economics, its analysis of human behavior (i.e., purchasing) and cognition (i.e., decision-making and attention) firmly situates this study within our sphere of interest. By using BONDS, Lacetera and colleagues (2012) were able to demonstrate the power of decision-making heuristics even within very real and high-stakes contexts: Left-digit biases cost buyers hundreds of dollars on a long-term purchase. The study shows the impact of cognitive biases at a scope that would be functionally impossible in laboratory research.

#### Sequential dependencies in online reviews (Vinson, Dale, & Jones, 2016)

Vinson and colleagues leveraged 2.2 million online business reviews posted on Yelp (http://www.yelp.com) to understand how sequential dependence functions in higher-order, real-world cognition. *Sequential dependence* (SD) is the phenomenon of earlier judgments affecting later ones, by making the later judgments either more similar (*assimilation*) or less similar (*contrast*) to the earlier ones. SD had largely been studied in psychophysiological research, such as in auditory or visual perception. Vinson and colleagues extended this to higher-level and more complex settings by looking at each review’s positivity or negativity (measured in 1–5 “star” ratings) relative to that reviewer’s previous reviews. They found evidence that SD does, indeed, affect this complex cognitive process at both the individual and group levels: Individual reviewers tended to show contrast SD effects, whereas reviews of the same business by multiple individuals tended to show assimilation SD effects across shorter timescales (measured in days).

By taking advantage of Yelp as BONDS, Vinson and colleagues (2016) were able to show the real but subtle effects of SD on behavior and cognition outside the lab. Because higher-order cognitive processes—like those underpinning business reviews—are complex, it would be difficult to identify the slight nudge by previous reviews on any current review without large-scale, messy, highly variable data. These results show that the cognitive biases that can be prominently identified in simple lab tasks can also impact our everyday behavior—including the public perceptions of businesses.

## The gaps

Cognitive scientists and psychologists often have a vague sense of excitement when talking about these new data opportunities, but this enthusiasm has not yet led to the broad adoption of BONDS. A variety of reasons have led to this lag, which can broadly be categorized into three “gaps”—the imagination gap, the skills gap, and the culture gap.

The three gaps—although daunting—are not insurmountable, and researchers in our field have incredible strengths derived from experimental training that can serve them well in BONDS research. Cognitive science and psychology training emphasizes theory-grounded training with strong inferential and critical-thinking skills. Solutions, then, should be specifically engineered to leverage the field’s existing strengths while bridging the gaps where BONDS efforts have moved beyond the field’s current training and mindset.

Toward that end, we created the website Data on the Mind (http://www.dataonthemind.org), home to a new community-focused initiative to help cognitive scientists and psychologists use BONDS to understand behavior and cognition. Our goal is to help bridge the three gaps within the context of theory-building research and emerging ethical issues. Data on the Mind is fundamentally designed to specifically target the strengths and needs of the cognitive science and psychology community. We welcome involvement by fellow researchers—whether by pointing out new resources or suggesting new ways that we can help meet the community’s needs.

Below we outline how we see each of these gaps as being bridged, along with preliminary steps that we have taken toward doing so through establishing Data on the Mind.

### The imagination gap

The imagination gap is the inability of researchers to see themselves, their research area, and their specific research question in the BONDS around them. This gap may not be the most immediately striking one, but it is one of the most functionally limiting. Today’s researchers know that companies, governments, and other organizations are capturing massive amounts of data. However, from our conversations with interested researchers, very few can envision a dataset that would address an important theoretical question in their field—let alone know where to start looking for one. This is especially true for researchers who do not deal with language, given that most high-profile BONDS are linguistic (e.g., social media content).

Bridging the imagination gap will take some work to adjust our field’s idea of the possible scope of data beyond experimentally generated datasets. This requires the curiosity to continue hunting down new possible datasets, the theory-guided creativity to see their potential, the ethical constitution to critically question their use, and the willingness to share with others.

Even today, a wealth of data can be used to address a variety of research areas. Language-centric data abound, from decades of transcripts from U.S. federal congressional hearings (https://www.gpo.gov/fdsys/browse/collection.action?collectionCode=CHRG) to the entirety of Wikipedia (Wikimedia Foundation; https://dumps.wikimedia.org/). Researchers interested in understanding categorization might investigate tagging behavior in the Yahoo Flickr Creative Commons 100M dataset (http://webscope.sandbox.yahoo.com/catalog.php?datatype=i&did=67; Thomee et al., 2016). Decades of online chess game records could shed light on expertise and decision making (e.g., Free Internet Chess Server Database; http://www.ficsgames.org/download.html), and play-by-play sports records might be useful for studying team dynamics (https://www.kaggle.com/maxhorowitz/nflplaybyplay2015). Government data can also provide new avenues for research: With U.S. cities and states from Nashville (https://data.nashville.gov/) to New York (https://data.ny.gov/) embracing data transparency, researchers can weave together multiple data records to explore complex patterns of behavior and cognition in everyday life. Goldstone and Lupyan (2016) provide an excellent table with many more examples of research questions and suggestions for relevant datasets.

**Fig. 1:**
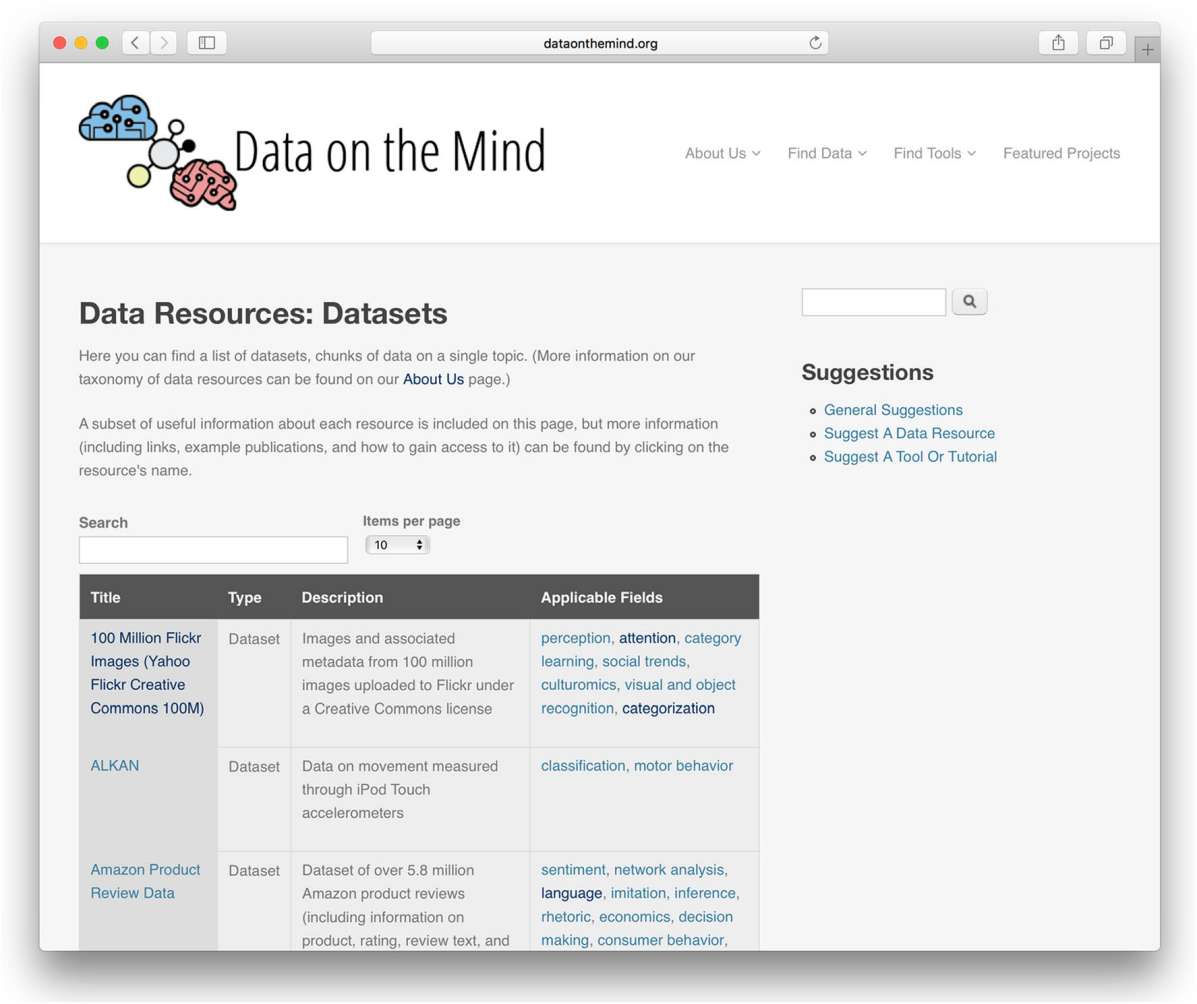
Screenshot of curated datasets from Data on the Mind (http://www.dataonthemind.org/data-resources/datasets), a branch from our list of data resources (http://www.dataonthemind.org/find-data). Each entry in this table includes the name of the data resource, a brief description, and the research area(s) in cognitive science and psychology it could be relevant to. Further information (including where to find the data and what is required to access them) is available by clicking on the name of the dataset

To address the imagination gap, Data on the Mind curates lists of BONDS to specifically address different research areas (see Fig. 1). Each entry is labeled with one or more relevant area(s) of study, such as attention, categorization, decision making, or language acquisition—research areas at the level of an introductory psychology textbook. All resources are specifically chosen because of their out-of-the-box cognitive or behavioral potential: While perhaps not created for research purposes, these resources present ripe opportunities for uncovering principles of human behavior and cognition. In this way, we hope to create an easily accessible repository to help spark the imagination.

### The skills gap

The skills gap is perhaps the most obvious of the three. Given the power and scope of these new data, researchers may ask themselves what new tools, methods, and analyses are needed to make sense of them. Many BONDS are too large to be opened in standard spreadsheet software (assuming that the dataset is even in a spreadsheet format), too messy to be analyzed upon collection, and too complex to be appropriately analyzed by simple inferential statistics.

Skills like database management, data procurement (e.g., using APIs, web scraping), data “munging” (i.e., cleaning), and scientific programming are essential to BONDS research but are not often taught in traditional undergraduate and graduate courses in our field. The best way to bridge this gap lies in creating training opportunities that are targeted at the specific strengths and weaknesses of researchers in cognitive science and psychology. These training opportunities should be grounded within the framework of the overarching research area: What works best for a computer science graduate student will likely not be best for a psychology graduate student.

Addressing the skills gap will take more time and effort than addressing the imagination gap. A wealth of training materials for basic programming exists through massive open online courses (MOOCs) and online tutorials, but these are often taught for and by computer scientists. These can provide excellent jumping-off points for researchers from any domain, but cognitive science and psychology must begin creating workshops, summer schools, and formal education programs to equip researchers at *every* career stage to effectively use BONDS for theory-driven research.

**Fig. 2:**
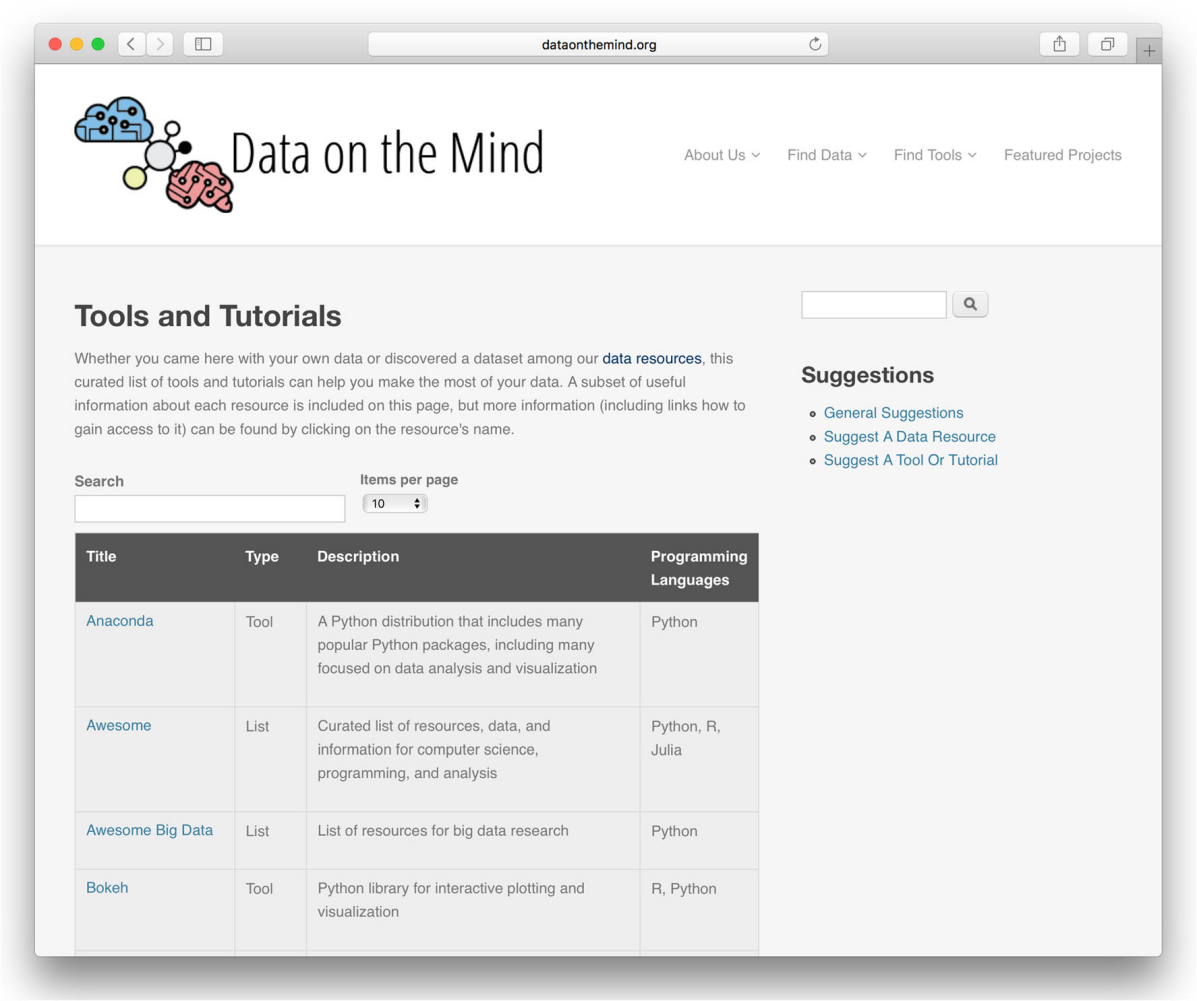
Screenshot of curated tools and tutorials from Data on the Mind (http://www.dataonthemind.org/tools-and-tutorials), a branch from our list of all such resources (http://www.dataonthemind.org/find-tools). Each entry in this table includes the name of the resource, a brief description, and its programming language(s). Further information (including where to find the tool) is available by clicking on the name of the resource

To address the skills gap, Data on the Mind identifies tutorials and tools that will help researchers in our field handle BONDS (see Fig. 2). Resources like Coursera (https://www.coursera.org) and Khan Academy (https://www.khanacademy.org) are available to learn basic skills, but we believe that the most effective solutions to the skills gap will be tailored to complement traditional training efforts. By putting together our own tutorials and curating existing ones, we aim to provide researchers with skills and tools that can supplement their existing strengths.

### The culture gap

The *culture gap* is the difficulty in getting the BONDS perspective adopted by individuals and institutions within cognitive science and psychology (and getting the holders of these datasets, which are often technology companies, to recognize the value that cognitive science and psychology have to offer in analyzing their data). The difference between interest in BONDS research and utilizing BONDS in research can be partially attributable to a lack of role models and acceptance of these new data resources. The subtlety and pervasiveness of this gap makes it the least obvious and the hardest to address outright.

Although working to bridge the imagination and skills gaps will undoubtedly help close the culture gap, efforts to raise the profile of theory-driven BONDS research within cognitive science and psychology will be essential. Journal editors, for instance, could help mediate between reviewers—who may not have performed or read BONDS research before—and authors, giving authors greater opportunity to address criticisms of both BONDS research broadly and their specific manuscript. Departments might help by developing coursework at the intersection of BONDS and traditional research methods, possibly by teaming up with computer science departments. Perhaps most importantly, researchers *actively engaged in* BONDS work should consider ways that they can contribute to changing the community through outreach, such as teaching workshops, participating in conference panels, and online venues (e.g., social media, blogs).

**Fig. 3:**
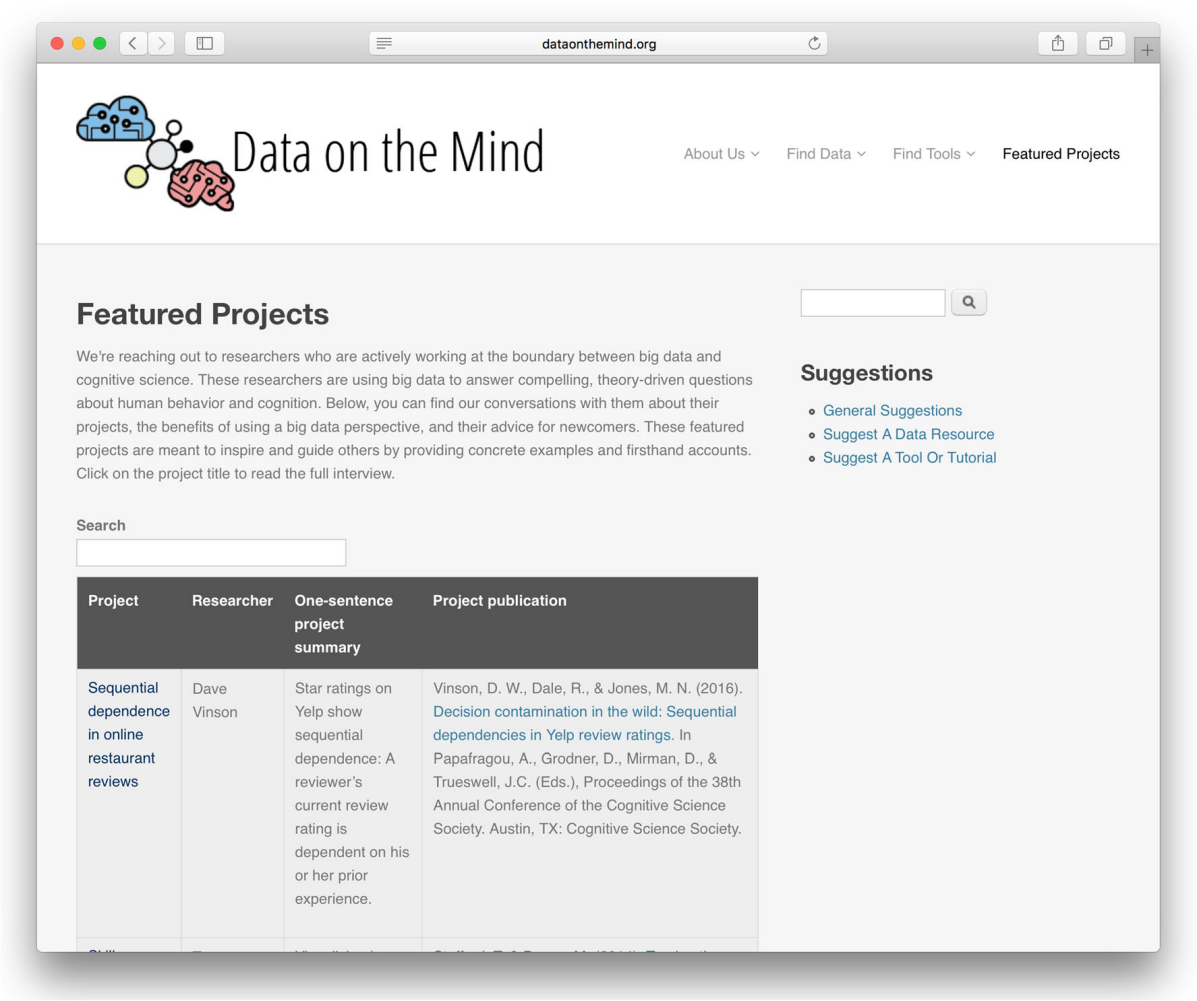
Screenshot of Data on the Mind’s table of interviews with researchers in cognitive science and psychology about their theory-driven research using BONDS (http://www.dataonthemind.org/featured-projects). Each entry includes the name and a synopsis of the project, the researcher, and a publication reference. The extended interview is available by clicking on the project name

To address the culture gap, Data on the Mind provides resources to help educate researchers about the BONDS perspective. We are currently focusing our efforts on highlighting researchers in cognitive science and psychology who are pioneering theory-driven research using BONDS (see Fig. 3). Although many researchers have extensive experience with laboratory experiments, very few know how to navigate research in this new frontier. These project-focused interviews with active researchers will help provide inspiration and practical advice for others interested in BONDS research, giving them essential insights into the feeling of performing this research. They also provide examples of scientific impact that are informative for companies and other holders of potentially relevant datasets.

## Concerns about ethics and responsible science in BONDS

Questions of ethics in BONDS research stand as one of the most pressing concerns facing cognitive science and psychology. As was noted relatively early in the data revolution in science (Boyd & Crawford, 2012), simply having access to data does not confer a blank check for their use. Scientific and lay communities have engaged in serious discussions about ethical guidelines following some highly publicized studies over the last several years (e.g., Kirkegaard & Bjerrekær, 2016; Kramer, Guillory, & Hancock, 2014). Establishing ethical norms for the use of BONDs, then, is critical to the future of the scientific community’s relationship with the broader public—both in keeping with our responsibility to the public as data creators (or users) and in maintaining the public’s trust in science.

From journalism (Fairfield & Shtein, 2014) and education (Willis, Campbell, & Pistilli, 2013) to psychology (Fiske & Hauser, 2014; Puschmann & Bozdag, 2014) and artificial intelligence (Russell, Dewey, & Tegmark, 2015), a range of fields are grappling with issues regarding how to reconcile these new data with our duties to the public. For example, Hovy and Spruit (2016) recently staked out a variety of issues in natural-language processing (NLP) and machine-learning research, pointing out the implications for individuals and society at the intersection of NLP and social media. These articles make powerful cases regarding the potential damages to individual participants (including the real impact of analysis over personally identifiable information), to scientific and industrial products based on the data (including the perpetuation of systemic and/or institutionalized bias), and to society at large (including mistrust of scientists and misunderstanding of the scientific process).

These problems cannot be solved simply on the “supply side” of the data, like including waivers in terms of service. Not only do the overwhelming majority of consumers fail to fully read such documents (Obar & Oeldorf-Hirsch, 2016), but putting the onus on participants rather than researchers runs counter to the spirit of informed consent. Instead, the “use side” of the data must be vigilant against possible misuses of data, even after data have been collected. The researcher’s ethical responsibility should encompass the entire lifecycle of BONDS, commensurate with the importance of open science and reproducibility.

### Responsibility to the public as participants

The scientific community must move forward with creating a code of ethics that governs such research through robust conversations with the wider lay and scientific communities. In the meantime, the principles laid out in the Belmont Report (National Commission for the Protection of Human Subjects of Biomedical and Behavioral Research, 1978) can continue to provide guidance to researchers. Although later superseded by the so-called “Common Rule” (U.S. Department of Health and Human Services, 1991), the Belmont Report’s three fundamental principles for ethical treatment of subjects or participants remain highly influential: respect for persons (i.e., acknowledging and respecting participant autonomy), beneficence (i.e., maximizing benefits and minimizing risks for participants), and justice (i.e., recruiting participants fairly and equally from as broad a sample of the population as possible, without exploitation or favoritism).

Today, our community must expand the breadth of these principles. Respect for persons should inform the ways in which researchers decide to mine and use online data. Beneficence and justice should lead to an increased awareness of analyzing and publishing data about individuals—even seemingly innocuous data (e.g., Ramakrishnan, Keller, Mirza, Grama, & Karypis, 2001)—in a time during which digital records persist almost indefinitely; even some data claimed to be anonymized can be leveraged to find sensitive information (cf. Netflix data; Narayanan & Shmatikov, 2008). The same concern that researchers have for participants in their labs should extend to those they may never meet, with special attention to 21st-century risks.

### Maintaining the public’s trust in science

Because conflicts among scientists erode public trust in science in the United States (Nisbet, Cooper, & Garrett, 2015; this may not hold for other countries, though: cf. Andersson, 2015), recent concerns over reproducibility in psychology (e.g., Open Science Collaboration, 2015) and other scientific fields (e.g., Gezelter, 2015; Peng, 2015) are making transparency and openness increasingly important. Openness, then, is a particularly timely advantage of using BONDS, given the growing availability of freely accessible data. When tapping into freely available data, researchers must only publish their complete code at an open repository (e.g., GitHub <https://www.github.com> or the Open Science Framework <https://osf.io>) to allow a fully reproducible and transparent workflow.

### Addressing ethics

These dual concerns are, of course, complicated and highly interconnected. Two high-profile examples in the past few years involved the use of data from Facebook (Kramer et al., 2014) and from a dating website called OKCupid (Kirkegaard & Bjerrekær, 2016). In both cases, the public and the scientific community raised concerns over issues of informed consent, participant privacy, and transparency. As BONDS become more widely utilized in scientific research, resolving these ethical issues will be imperative to maintaining the public’s trust in the scientific process.

While above we have laid out some ideas to bridge the three gaps, these complex ethical issues remain unsolved. These are the kinds of issues that we are thinking about how to handle next in the context of Data on the Mind. By bringing together diverse perspectives, we hope to come to solutions that will prioritize ethical protections and public concerns within the scientific process.

## Conclusion

The use of big data and naturally occurring datasets provides unprecedented opportunities and challenges for understanding human behavior and cognition. These challenges—what we call the *imagination gap*, the *skills gap*, and the *culture gap*—are situated within ongoing questions about ethics and scientific responsibility. Meeting these challenges will require community engagement and investment—which are well worth the benefits to theory-building afforded by data at a previously unthinkable scale. Rather than supplanting laboratory investigations, research using BONDS can supplement traditional approaches by serving as a proving ground for theories developed in rigorously controlled experiments. We welcome others to join us in using, developing, and promoting BONDS efforts, whether through Data on the Mind or through new initiatives. Ultimately, we see any expansion of this area as a much-needed step toward integrating BONDS that speak to cognitive science and psychology into our theoretical toolkit.
